# Promoter G-quadruplex folding precedes transcription and is controlled by chromatin

**DOI:** 10.1186/s13059-021-02346-7

**Published:** 2021-05-07

**Authors:** Jiazhen Shen, Dhaval Varshney, Angela Simeone, Xiaoyun Zhang, Santosh Adhikari, David Tannahill, Shankar Balasubramanian

**Affiliations:** 1 Li Ka Shing Centre, Cancer Research UK Cambridge Institute, Robinson Way, Cambridge, CB2 0RE UK; 2 Yusuf Hamied Department of Chemistry, University of Cambridge, Cambridge, CB2 1EW UK; 3 School of Clinical Medicine, University of Cambridge, Cambridge, CB2 0SP UK

**Keywords:** G-quadruplexes, Transcription, RNA polymerase II, Chromatin compaction

## Abstract

**Background:**

Four-stranded G-quadruplexes (G4s) are DNA secondary structures in the human genome that are primarily found in active promoters associated with elevated transcription. Here, we explore the relationship between the folding of promoter G4s, transcription and chromatin state.

**Results:**

Transcriptional inhibition by DRB or by triptolide reveals that promoter G4 formation, as assessed by G4 ChIP-seq, does not depend on transcriptional activity. We then show that chromatin compaction can lead to loss of promoter G4s and is accompanied by a corresponding loss of RNA polymerase II (Pol II), thus establishing a link between G4 formation and chromatin accessibility. Furthermore, pre-treatment of cells with a G4-stabilising ligand mitigates the loss of Pol II at promoters induced by chromatin compaction.

**Conclusions:**

Overall, our findings show that G4 folding is coupled to the establishment of accessible chromatin and does not require active transcription.

**Supplementary Information:**

The online version contains supplementary material available at 10.1186/s13059-021-02346-7.

## Background

Four-stranded G-quadruplex (G4) structures form in DNA from stacked tetrads of Hoogsteen-bonded guanines [1]. G4 sequence motifs are prevalent at promoters in the human genome [2–4], and ChIP-sequencing using a G4 structure-specific antibody has revealed that endogenous G4s are enriched in nucleosome-depleted regions (NDRs) upstream of transcription start sites (TSSs) [5]. Genes marked by endogenous promoter G4s in chromatin show higher transcriptional output than their non-G4 counterparts [5]. Chromatin relaxation by histone deacetylase inhibitors can also lead to an increase in G4 formation [5]. In patient-derived aggressive breast cancer, promoters of highly amplified genes that show increased expression also exhibit increased G4 formation in chromatin [6]. As more than 99% of endogenous G4s overlap with transcription factor (TF) binding sites [7], it is possible that such elevated gene expression results from increased TF occupancy at promoter G4s and the recruitment of RNA Pol II. The observation that several TFs display high-affinity binding for G4s in vitro, including SP1 [8], CNBP [9], and LARK [10], lends support to this. Alternatively, TF recruitment to G4s could enhance transcription by unfolding the G4 structure, as has been suggested for CNBP [11]. On the other hand, torsional stress and negative superhelicity have been proposed to stimulate G4 formation at promoters as a consequence of active transcription [12].

A central unanswered question is whether G4 formation at promoters of active genes is a cause or a consequence of increased transcriptional activity. Herein, we establish that the folding of G4s in promoters does not necessarily require active transcription but can be favoured by an accessible chromatin state. Moreover, we provide evidence that G4s are a genomic feature that enables the recruitment of Pol II to promoters.

## Results

### G4 structures mark promoters with increased Pol II occupancy

**Fig. 1:**
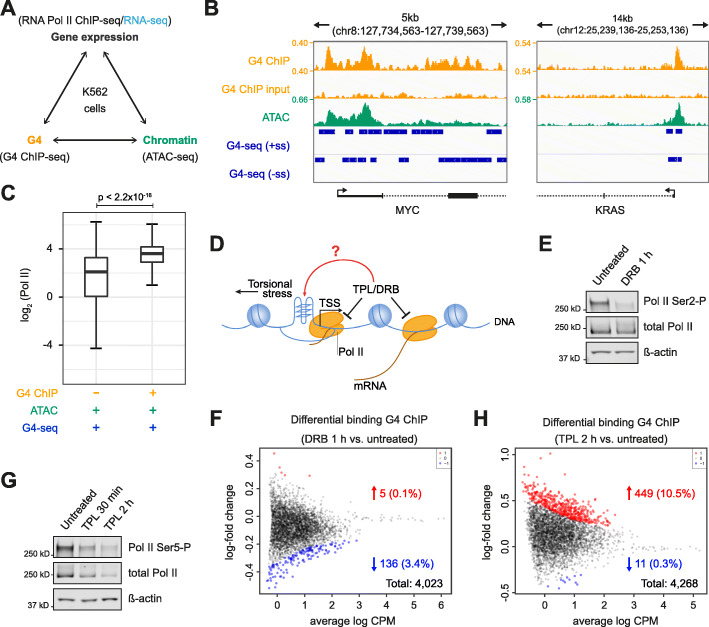
Transcription is not necessary for G4 formation in promoters. **a** Experimental design overview. Black, data generated in this study; blue, published RNA-seq datasets. **b** Representative examples of G4 ChIP-seq genomic tracks in K562 cells for *MYC* and *KRAS*. From top to bottom tracks show G4 ChIP-seq signal (yellow), G4 ChIP-seq input control (yellow), ATAC-seq signal (green) and sequence motifs that can form G4s in vitro (defined as G4-seq sites, see *ref* [15]) on the forward (+ss) or reverse (−ss) strand (blue). The signal is quantified as count per million (CPM). **c** Box plot of Pol II ChIP-seq signal (log_2_CPM) at promoters (TSS ± 500 bp) in accessible chromatin (ATAC +) with an endogenous G4 (G4 ChIP +) or without a G4 (G4 ChIP−) but having sequence motifs that can fold into G4 structures in vitro (G4-seq). Wilcoxon-test: *p* < 2.2 × 10^− 16^. **d** Graphical representation of G4 formation and transcriptional inhibition experiments. **e** Western blotting for Pol II Ser2-P and total Pol II from K562 cells treated with or without 100 μM DRB for 1 h. ß-actin provides the loading control. **f** Bland Altman (MA) plot showing the fold change in G4 ChIP-seq signal at promoters between DRB-treated versus DMSO-treated K562 cells. Statistically significant (*p* < 0.01) higher and lower signals are in red and blue, respectively; black dots indicate regions not changing. **g** Western blotting for Pol II Ser5-P and total Pol II for K562 cells treated with or without 10 μM triptolide (TPL) for 30 min or 2 h. ß-actin provides the loading control. **h** MA plot similar to panel F illustrating the fold change in G4 ChIP-seq signal at promoters between TPL-treated versus untreated K562 cells (*p* < 0.01)

To investigate whether the process of transcription modulates G4 structure formation in chromatin, we used the extensively characterised K562 human chronic myelogenous leukaemia cell line as a model system [13]. G4 structure formation was determined by G4 ChIP-seq [14], chromatin accessibility by ATAC-seq (assay for transposase-accessible chromatin using sequencing) and Pol II occupancy by Pol II ChIP-seq (Fig. 1a). Consensus, endogenous G4s were defined as G4 ChIP-seq peaks present in at least two out of three biological replicates (Pearson’s correlation *R* > 0.96). The majority of the consensus G4 peaks (> 75%) comprise G4 sequence motifs that have been independently shown to fold into a G4 structure in vitro, by a DNA polymerase stalling assay (G4-seq; Additional file 1: Fig. S1A) [15]. In K562 cells, consensus G4s were highly enriched in promoters (defined as TSS ± 500 bp; Additional file 1: Fig. S1B), hereafter referred to as promoter G4s. Promoter G4s marked genes with significantly increased expression compared to promoters lacking a G4 structure (*p* < 2.2 × 10^− 16^; Additional file 1: Fig. S1C; RNA-seq dataset GEO accession no. GSE88473). ATAC-seq on three independent biological replicates (Pearson’s correlation *R* > 0.98) further revealed that the majority of all consensus G4s (88.2%) were located in NDRs (Additional file 1: Fig. S1D) as exemplified by *MYC* and *KRAS* promoter G4s (Fig. 1b). These results corroborate our previous observations, in HaCaT cells and primary keratinocytes [5] and support that endogenous G4s are primarily found within open chromatin at promoters. Given that endogenous promoter G4s mark elevated transcription, as a first step, we determined Pol II occupancy at such genes. Using Pol II ChIP-sequencing (5 biological replicates; Pearson’s correlation *R* > 0.99), we observed significantly higher Pol II occupancy at promoters with an endogenous G4 compared to those without (*p* < 2.2 × 10^− 16^; Fig. 1c). To elucidate how G4 formation may be influenced by active transcription and whether G4 formation is promoted by a more open versus compacted chromatin environment, we have focused the majority of the following studies exclusively on promoter G4s (TSS ± 500 bp) occupied by Pol II.

### Promoter G4 formation does not require active transcription

We directly evaluated whether active transcription is required for the formation of promoter G4s, as has been suggested [12, 16], by measuring whether inhibition of transcription causes loss of G4s (Fig. 1d). To inhibit transcription elongation, K562 cells were treated with the CDK9 inhibitor 5,6-dichlorobenzimidazole 1-β-d-ribofuranoside (DRB) to prevent paused Pol II release [17, 18]. DRB treatment (1 h) decreased phosphorylation of Pol II at serine 2 with no effect on overall Pol II levels, as seen by Western blotting (Fig. 1e), confirming the expected inhibitory mechanism. The DRB-treated cells and DMSO-treated controls were subjected to G4 ChIP-seq. Promoter G4s in DRB- versus DMSO-treated cells did not show statistically significant changes, in the G4 ChIP-signal at the majority of sites (*p* < 0.01; 5/4023, 0.1% up, 136/4023, 3.4% down; Fig. 1f). Thus, inhibition of Pol II-dependent elongation does not lead to loss of G4 formation at promoters, rather G4 folding at promoters must precede productive transcription elongation.

To evaluate whether inhibition of transcription initiation causes loss of promoter G4s, we used triptolide (TPL), which covalently inhibits the Pol II-associated helicase XPB [19]. As seen by Western blotting, 2 h TPL treatment leads to substantial loss of Pol II serine 5 phosphorylation and an overall loss of Pol II protein levels (Fig. 1g). However, TPL inhibition did not significantly decrease the overall promoter G4-ChIP signal (*p* < 0.01; 11/4268 down, 0.3%; Fig. 1h). Conversely, a significant number (*p* < 0.01; 449/4268 up, 10.5%) of promoter G4s showed increased G4 signal after TPL treatment. This contrasts with experiments in human adenocarcinoma cells that showed no G4 alterations following TPL treatment [20]. Our observation of increased G4 formation at promoters following TPL treatment is likely to arise from the abrogation of the helicase activity of the G4-resolving helicases XPB/XPD [19, 21]. In contrast to earlier work suggesting that G4 folding is promoted by active transcription through the generation of single-stranded DNA [12], our results suggest that active transcription is not necessary for the folding of G4s in promoters.

### Endogenous promoter G4 folding is sensitive to chromatin compaction

**Fig. 2:**
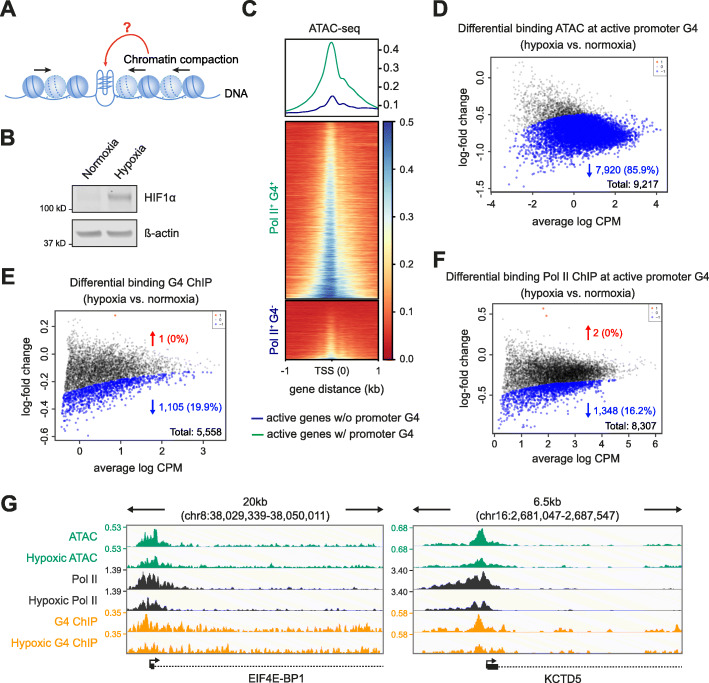
Chromatin compaction diminishes promoter G4 folding. **a** Graphical representation of experimental design examining G4s, transcription and chromatin compaction induced by hypoxia. **b** Western blotting for HIF1α from K562 cells cultured under normoxic or hypoxic conditions. ß-actin provides the loading control. **c** Chromatin accessibility at promoters under normoxic conditions. Top panel, metagene plot for the median of the normalised ATAC signal (3 biological replicates) centred at the TSS under normoxic conditions. Green, promoters of active genes with a G4 (Pol II^+^ G4^+^); blue, active genes without a promoter G4 (Pol II^+^ G4^−^). Bottom panel, data plotted for individual loci and represented by heatmaps. **d–f** MA plots showing fold change in ATAC-seq signal (**d**), G4 ChIP-seq signal (**e**) and Pol II ChIP-seq signal (**f**) following hypoxia at active promoters with a G4 (Pol II^+^ G4^+^). Blue and red, sites with significantly reduced or increase signal respectively (*p* < 0.05). CPM read count per million. **g** Genomic view of *EIF4E-BP1* and *KCTD5* exemplify genes that significantly change in ATAC-seq (green), Pol II ChIP-seq (black) and G4 ChIP-seq peaks (yellow). Tracks compare peaks between hypoxia and normoxia. Genomic coordinates indicate track range and the signal is quantified as counts per million (CPM). In all panels normoxia refers to cells cultured in 21% O_2_ and hypoxia refers to cells exposed to 1% O_2_ for 1 h

Given transcriptional inhibition does not remove promoter G4s in chromatin, and since chromatin relaxation by histone deacetylase inhibition can increase their formation [5], we hypothesised that the chromatin state may regulate promoter G4 formation. To explore this, we used hypoxia to manipulate the chromatin state (Fig. 2a). Hypoxia induces global chromatin compaction [22, 23] characterised by increased heterochromatin protein HP1BP3 expression [24], elevated histone H3 lysine 9 methylation (H3K9me3) [25] and reduced histone acetylation [26]. K562 cells were exposed to acute hypoxic conditions (1% oxygen, 1 h). Hypoxia was confirmed by Western blotting for hypoxia-inducible factor 1α induction [27] (Fig. 2b). Genome-wide chromatin compaction upon hypoxia was then demonstrated by decreased sensitivity to micrococcal nuclease digestion [28] (Additional file 1: Fig. S2A). Moreover, in hypoxic chromatin, ATAC-seq showed increased fragment sizes consistent with chromatin compaction (Additional file 1: Fig. S2B). Additional validation of hypoxia induction was given by decreased Pol II occupancy seen at genes known to have reduced expression in hypoxic K562 cells [29] (Additional file 1: Fig. S2C).

We first determined the chromatin status of active gene promoters in normoxia (21% O_2_) by ATAC-seq and found that G4-marked promoters (i.e. Pol II^+^ G4^+^) are located in more accessible chromatin compared to their active non-G4-marked (i.e. Pol II^+^ G4^−^) counterparts (Fig. 2c). The induction of hypoxia then resulted in a significant reduction in ATAC-seq signal intensity at the majority of G4 promoter sites (*p* < 0.05; 7920/9217 down, 85.9%; Fig. 2d). In comparison, non-G4-marked promoters show much less chromatin compaction (Additional file 1: Fig. S2D). After induction of hypoxia, about a fifth of the promoter G4s showed a statistically significant decrease in signal intensity (*p* < 0.05; 1105/5558 down, 19.9%; Fig. 2e), which indicates that many promoter G4s are sensitive to chromatin compaction. We next validated the general principle of G4 loss upon hypoxia-associated chromatin compaction by using an additional unrelated cell line. Acute hypoxia in human osteosarcoma U2OS cells also induced chromatin compaction and led to a reduction of G4 signal intensity at promoters (*p* < 0.05; 6150/8505 down, 72.3%; Additional file 1: Fig. S3).

In hypoxic K562 cells, we also observed a significant loss of overall Pol II signal intensity at Pol II^+^ G4^+^ promoters (*p* < 0.05; 1348/8307 down, 16.2%; Fig. 2f), with Pol II loss occurring almost entirely at sites where G4s were diminished (Additional file 1: Fig. S2E). These findings are exemplified by genome browser views in Fig. 2g. Conversely, there was negligible Pol II loss at Pol II^+^ G4^−^ promoters (Additional file 1: Fig. S2F). Thus, chromatin compaction causes a loss of many promoter G4s with concomitant loss of Pol II.

### G4 stabilisation by small molecules counteracts G4 and Pol II loss in hypoxia

To directly assay if G4 stabilisation impacts G4 formation in hypoxia, we visualised nuclear G4 foci [30] using U2OS cells since K562 cells were not amenable to G4 imaging. pyPDS, a PDS analogue with improved lipophilicity (clogP PDS 2.35, pyPDS 4.79, calculated using MarvinSketch version 20.19.0), was used to stabilise G4s [31, 32] (Additional file 1: Fig. S4A). In hypoxia without pyPDS, global G4 signal intensity and G4 foci numbers were reduced (*p* < 0.0001, Additional file 1: Fig. S5). However, with pyPDS addition, fewer G4s were lost (*p* < 0.0001), showing that G4 stabilisation protects G4s from unfolding in hypoxia.

**Fig. 3:**
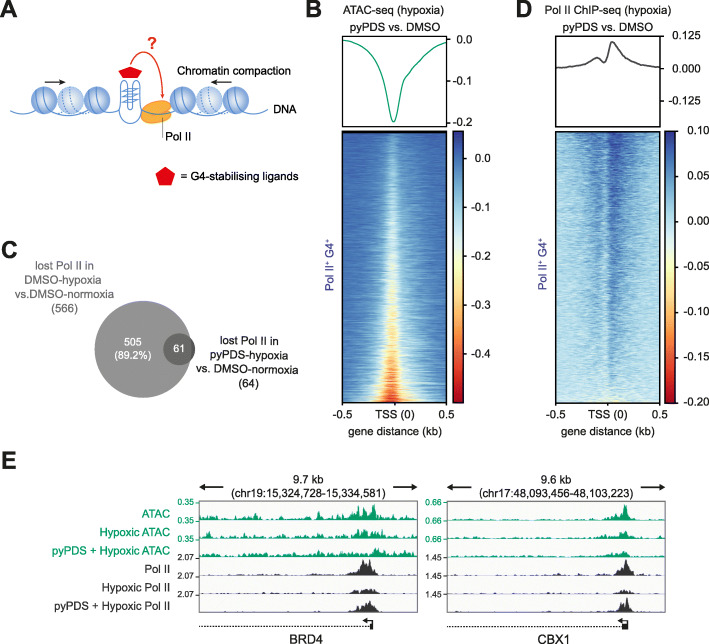
G4 stabilisation in compacted chromatin retains promoter Pol II occupancy. **a** Graphical representation of experimental design examining the consequences of G4 stabilisation by pyPDS on chromatin compaction and Pol II occupancy. **b** Chromatin accessibility differences for promoters of active genes with a G4 (Pol II^+^ G4^+^) under hypoxia for cells treated with DMSO or pyPDS. Top panel, metagene plot of ATAC signal centred at the TSS showing the signal difference between DMSO- and pyPDS-treated hypoxic cells. Bottom panel, data plotted for individual loci and represented by a heatmap plot. **c** Venn diagram showing sites that have lost Pol II occupancy between normoxic and hypoxic cells treated with DMSO and its overlap with Pol II sites lost on treatment with pyPDS in hypoxic conditions compared to DMSO treatment in normoxic conditions. This shows that the majority of Pol II sites seen reduced in hypoxia are retained by pyPDS. **d** Similar to panel **b** but for signal differences in Pol II occupancy. **e** Genome browser view displaying examples of genes involved in chromatin biology (e.g. *BRD4* and *CBX1*) that show changes in chromatin accessibility (ATAC-seq, green) and Pol II occupancy (Pol II ChIP-seq, black) for cells under normoxic (top), hypoxic (middle) or hypoxic conditions treated with pyPDS (bottom). Genomic coordinates are indicated above and signal quantified as counts per million (CPM)

Given that chromatin compaction leads to a loss of Pol II at sites where promoter G4s are diminished, we evaluated whether the induced persistence of G4 structures in hypoxia caused by G4 stabilisation could cause retention of RNA Pol II binding (Fig. 3a). Using ATAC-seq with K562 cells, we first confirmed that pyPDS treatment did not appreciably alter chromatin accessibility in normoxia and that chromatin compaction still ensues under hypoxic conditions (Fig. 3b, Additional file 1: Fig. S4B). For Pol II^+^ G4^+^ promoters, we found that pyPDS treatment reduced the loss of Pol II by 10-fold in hypoxia compared to DMSO-treated cells (10.6% vs 1.2% respectively, *p* < 0.05; Fig. 3c, d; Additional file 1: Fig. S4C-D). These findings are exemplified by genome browser views for the promoters of the chromatin regulators *BRD4* and *CBX1* (Fig. 3e). Furthermore, no changes in Pol II were observed with pyPDS treatment in normoxia (Additional file 1: Fig. S4E), or at non-G4-marked promoters in pyPDS-treated hypoxic cells (Additional file 1: Fig. S4F). These results rule out non-specific ligand-associated effects on Pol II recruitment. Thus, the induced persistence of a G4 structure, that would otherwise be lost during chromatin compaction, causes retention of Pol II binding.

## Discussion

**Fig. 4:**
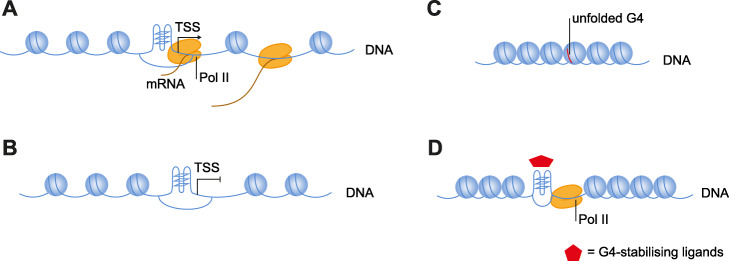
Model showing an interaction between promoter G4s, Pol II and chromatin status. **a** Promoter G4 folding is seen in accessible promoter regions. Pol II is recruited to the G4 to promote transcription. **b** Transcriptional inhibition does not cause loss of promoter G4 folding. **c** Chromatin compaction causes G4 unfolding and loss of Pol II binding. **d** Ligand-mediated G4 stabilisation (red star) can preserve promoter G4 folding under conditions of chromatin compaction, which in turn causes retention of Pol II

Here, through the use of chemical intervention, we show that the process of active transcription is not necessary for promoter G4 folding in cells (Fig. 4a, b). This suggests that negative superhelicity and strand separation of the DNA double helix associated with actively transiting RNA polymerase complexes is not necessary for G4 folding.

Instead, we find that changes in the chromatin environment are able to influence how G4s fold. In particular, by reducing chromatin accessibility, we can disrupt promoter G4s folding (Fig. 4c). The precise details of how G4s become unfolded during chromatin compaction under hypoxia are not clear. G4s have been suggested to be lost through abrogation of APE1 activity, a protein thought to drive G4 formation during base excision repair [20]. Hypoxia is associated with activation of DNA damage response [33], but importantly, we found no changes in APE1 protein levels during hypoxia either in the absence or presence of pyPDS (Additional file 1: Fig. S4G). This suggests that G4 loss during hypoxia is through an alternative mechanism.

We use acute hypoxia to achieve rapid chromatin compaction with a short treatment time to circumvent non-specific or downstream effects on G4 formation. Endogenous G4 structures are only observed in accessible chromatin, while chromatin relaxation by histone deacetylation leads to increased G4 folding [5]. Recent imaging experiments in neuronal cells have also demonstrated that G4 folding requires chromatin accessibility [34]. While we cannot rule out unknown factors that might further influence G4 formation in hypoxia, the most straightforward interpretation of the data is that chromatin compaction in acute hypoxia leads to G4 loss.

Concurrent with promoter G4 loss during chromatin compaction, we find that Pol II occupancy is also lost at the same promoters. We observe that if we apply a G4-specifc small molecule ligand to stabilise promoter G4s, Pol II loss is abrogated. Thus, promoter G4s and Pol II occupancy are coupled. This experiment also demonstrates that G4s can be manipulated to augment Pol II occupancy in an otherwise inhibitory chromatin environment (Fig. 4d). Pol II recruitment at promoter G4s might be mediated by TF binding [8–10], though it is also possible that G4s interact directly with subunits of RNA Pol II [35].

## Conclusions

In conclusion, our findings directly demonstrate that it is chromatin status and not transcription that is a primary determinant of promoter G4 folding in cells. Furthermore, promoter G4 folding leads to the retention of RNA Pol II suggesting that G4s act as a site for the recruitment of key components of the transcriptional machinery. Our findings thus provide a possible mechanism for enhanced transcription of genes carrying a promoter G4, as the G4 structure itself may be sufficient to direct transcription.

## Methods

### Cell culture

K562 cells (ATCC, CCL-243) were cultured in RPMI 1640 (Thermo Fisher, 21875-034) supplemented with 10% of foetal bovine serum (FBS; Thermo Fisher, A3840402). U2OS cells (ATCC, HTB-96) were cultured in DMEM (Thermo Fisher, 41966-029) supplemented with 10% of FBS. Cells were cultured at 37 °C in 21% O_2_ and 5% CO_2_ unless stated otherwise. Cell line genotypes were certified by the supplier. Cells lines were confirmed mycoplasma-free by RNA capture ELISA. For hypoxia treatment, cells were incubated in 1% O_2_, 5% CO_2_ at 37 °C. 5,6-dichlorobenzimidazole 1-β-d-ribofuranoside (DRB) (Sigma), triptolide (Sigma) and pyPDS [31, 32] were dissolved in DMSO and used at a final concentration of 100 μM, 10 μM and 1 μM, respectively. For G4 stabilisation in hypoxia, cells were pre-treated with pyPDS for 1 h and exposed to 1% O_2_ in the presence of pyPDS for 1 h.

### Western blotting

Cells were washed twice with ice-cold PBS and lysed in Pierce® RIPA buffer (Thermo Fisher, 89900) by sonatication using a Bioruptor (Diagenode). The following primary antibodies were used for immunoblotting: RNA Pol II C-terminal domain (CTD) phospho Ser2 (Active Motif, 61084), RNA Pol II CTD phospho Ser5 (Abcam, ab5131), RNA Pol II CTD (Abcam, ab817), HIF 1α (BD Biosciences, 610958), β-Actin (CST, 4970 or Sigma, A5441) and APE1 (Novus Biologicals, NB100-116). IRDye secondary antibodies (LiCor) were used, and the blot was visualised using a LiCor Odessey CLx instrument.

### Micrococcal nuclease (MNase) digestion

One million cells were washed in ice-cold PBS and incubated in lysis buffer (10 mM Tris pH 8, 10 mM MgCl_2_, 0.5% NP-40, fresh protease inhibitors + 1 mM DTT) at 4 °C for 10 min. Nuclei were pelleted by centrifugation at 1400×*g* for 5 min at 4 °C and washed once with lysis buffer. Samples were digested with 0.5 U MNase at 25 °C in digestion buffer (15 mM Tris pH 7.5, 60 mM KCl, 15 mM NaCl, 250 mM Sucrose, 1 mM CaCl_2_, fresh PIC + 1 mM DTT) and the reaction stopped with an equal volume of MNase stop solution (40 mM EDTA + 0.5% SDS). DNA fragments were purified using QIAGEN MinElute kit. Equal amounts of DNA (300 ng) from each sample were then loaded and resolved on 2% E-Gel EX precast agarose gels (Thermo Fisher, G800802).

### G4 ChIP-Seq

G4 ChIP-Seq was performed with at least 3 biological replicates using the G4-specific antibody BG4 as described previously [14]. For each biological replicate, three independent technical replicates and matched inputs were sequenced (75 nt single-end) on an Illumina NextSeq instrument.

### RNA polymerase II (Pol II) ChIP-seq

RNA polymerase II (Pol II) ChIP-seq was performed essentially with 5 biological replicates as previously described [36]. Fifteen micrograms of chromatin was immunoprecipitated overnight with 5 μg RNA Pol II antibody (Abcam, ab817) bound to protein A/G sepharose (Thermo Fisher, 10002D). Sequencing libraries were prepared by using NEBNext® Ultra™ II DNA Library Prep Kit for Illumina (NEB, E7645) and sequenced (75 nt single-end) on an Illumina NextSeq instrument.

### ATAC-seq

ATAC-seq was performed essentially with 3 biological replicates as previously described [37]. Briefly, 50,000 K562 cells were collected and incubated with transposase Tn5 at 37 °C. After 1-h incubation, tagmented DNA samples were amplified using the Nextera Index kit (Illumina, FC-121-1030). DNA fractions were size selected and purified using AMPure XP beads (Beckman Coulter, A63880) according to the manufacturer’s instruction. Libraries were sequenced in paired-end with 75-nt read length using the Illumina NextSeq instrument.

### Immunofluorescence staining

Immunofluorescence staining with BG4 was performed essentially as described previously [30]. Digital images were taken using a TCS SP5 confocal microscope (Leica) with Zeiss Zen software and analysed with Icy [38]. One hundred to 200 nuclei were counted per condition. Frequency distribution graphs were plotted using GraphPad Prism (GraphPad Software Inc.).

### Human reference genome and relative genomic annotation

Human genome hg38 was downloaded from UCSC (hgdownload.cse.ucsc.edu/goldenPath/hg38/bigZips/hg38.fa.gz). Human annotations (gtf file) were downloaded from the Genecode project portal (ftp.ebi.ac.uk/pub/databases/gencode/Gencode_human/release_28/gencode.v28.annotation.gtf.gz, Release 28 GRCh38.p12). Annotations for genomic regions (i.e. exons, introns, intergenic regions, 3′UTR, 5′UTR and 58,381 promoters of all coding and not coding genes defined as TSS ± 500 bp) were extracted from the gtf file.

### ATAC-seq data analysis

Fastq reads were trimmed from adapters using cutatapt [39] (cutadapt -a AGATCGGAAGAGC -A AGATCGGAAGAG). Resulting reads were aligned to hg38 with bwa mem -M -t 12. Bam files were generated by using samtools view -Sb -F780 -q 10 -L (ver: 1.8) [40]. All libraries were sequenced twice and processed and aligned separately. Resulting alignments were merged and sorted. Duplicates were marked by Picard MarkDuplicates (ver: 2.20.3, http://broadinstitute.github.io/picard) and removed. Fragment size distribution was estimated using uniquely mapped reads bams with Picard CollectInsertSizeMetric. To assess the amount of mitochondria contamination, reads mapping to chrM were identified and counted directly from the alignment bam files. For each library, regions with local accessibility were identified by calling peaks with macs2 with default options and excluding chrM. For each experimental condition, peak regions observed in 2 out the 3 biological replicates were selected as the consensus regions using bedtools multiIntersectBed (version 2.27.1) [41].

### G4 ChIP-seq and Pol II ChIP-seq data analysis

Fastq reads were trimmed from adapters using cutadapt (-m 10 -q 20 -O 3 -a CTGTCTCTTATACACATCT) and aligned to the human genome hg38 with bwa mem. Bam files were generated from alignment with samtools view, and duplicated reads were marked and removed using picard MarkDuplicates. The total number of unique reads was quantified for each library. Regions with local enrichments were obtained by calling peaks with macs2 for each individual pull-down library paired to the corresponding input control. For G4 ChIP-seq experiments, consensus regions of each biological replicate were obtained as those observed in 2 out of the 3 technical replicates (multiIntersectBed). The consensus by the experimental case (across biological replicates) was obtained by selecting regions reproducibly observed in at least 2 of the 3 biological replicates. For Pol II ChIP-seq experiments, the consensus regions in each experimental condition were obtained as the regions observed in at least 3 of the 5 biological replicates.

For both types of ChIP-seq, genome-wide reads per million (RPM) G4 signal was obtained by quantifying the read coverage across the genome and scaling it to a factor that reflected the individual library size (deeptools bamCoverage [42]–scaleFactor, where factor = 1,000,000/Lib_size). Similarity across individual libraries from all three cell types was evaluated on RPM at consensus regions per experimental condition. Whenever it is referred to G4 or Pol II signal in a specific experimental condition, individual libraries of the same experimental condition were combined together by calculating the median RPM signal (across biological/technical replicates). G4 consensus regions were compared to the accessible consensus regions (ATAC) observed in the same experimental conditions and quantified in terms of percentage of overlaps; G4 consensus regions were compared to in vitro observed G4 quadruplex (G4-seq [43]) and quantified by evaluating the percentage of overlaps.

### Characterisation of G4 fold-enrichments at sites of interests

Fold enrichments for G4s over random chance were evaluated at various sites of interest whose genomic coordinates were stored into bed files. Fold enrichments were computed by using the Genomic Association Tester (GAT, https://gat.readthedocs.io/en/latest/contents.html, 1000 randomizations), and the analysis was restricted to the human whitelist. The analysis generated the number of overlaps between the G4 consensus sites and the segments to query against (actual), and the fold-enrichments were obtained after summarising results from randomisations.

### Density plots of genomic signals at TSS

The metagene density signal profile at transcription start sites (TSSs) was produced similarly for G4, Pol II and ATAC-seq data. After creating bed files with the set/s of regions of interest, we employed the function *computeMatrix* from deeptools on BW files containing RPM signals). Next, RPM signals were combined together by averaging replicates of the same cell and then generating a new matrix of signals. The difference of normalised summarised signals between two experimental conditions was obtained by subtracting the matrix of normalised signal in one condition to the matrix of the other condition under investigation. Heatmaps were produced using deeptools plotHeatmap (options: --averageTypeSummaryPlot median). Metagene density signal profile plots were obtained by plotting the average trend of the signal of interest (i.e. difference between two experimental cases).

### Differential signal analysis

All differential binding signal analyses were carried out with the R package edgeR. Initially, library size and read coverages at the regions of interest were computed. Prior differential testing, the average cpm (counts per millions) signal was estimated across all input libraries and a threshold value was defined as 2 times the 99th quantile of the average distribution of input cpm. Subsequently, regions for which at least one pull-down library exceeded the threshold value previously defined were kept for subsequent analysis. This step happened for each sequencing assay independently and only for the case when input libraries were available (G4 ChIP-seq, Pol II ChIP-seq). A generalised linear model (*glmLRT*) with default parameters (negative binomial log-linear distribution of read counts) was used to assess regions with a differential binding signal. For the differential test, batch information, biological and technical (when present) replicates were incorporated in the definition of the design matrix. The differential binding analysis compared pairs of experimental conditions: hypoxia vs normoxia (G4 ChIP-seq, ATAC-seq, Pol II ChIP-seq); all pairwise comparison among DMSO hypoxia, DMSO normoxia, pyPDS hypoxia and pyPDS normoxia. Regions with differential signal were identified as those with *p* value ≤ 0.05. The regions of interest used to test the effects of hypoxia plus pyPDS or hypoxia alone were defined as the G4 consensus regions overlapping Pol II consensus regions in normoxia at promoters (TSS ± 500 bp). In the case of DRB and TPL experiments, the regions used for the differential signal analysis were obtained as the merge of the regions observed in treatment and control case for each experimental condition, respectively. To illustrate the outcome of the differential analysis, Bland Altman (MA) plots showing the average CPM (*x*-axis) versus the log-fold change (*y*-axis) obtained comparing the 2 conditions of interest were used.

## Supplementary information

**Additional file 1: Fig. S1.** Endogenous G4 landscape in human K562 cells. **Fig. S2.** Induction of hypoxia decreases chromatin accessibility leading to G4 loss. **Fig. S3.** Hypoxia-induced chromatin compaction results in the reduction of promoter G4 folding in U2OS cells. **Fig. S4.** Response of endogenous G4s and Pol II following pre-treatment with G4-stabilising ligands before and after hypoxia induction. **Fig. S5.** BG4 immunofluorescence (IF) staining for G4s in DMSO- or pyPDS-treated cells upon hypoxia treatment.

**Additional file 2.** Uncropped Western blots.

**Additional file 3.** Review history.

## Data Availability

The data produced in this study (G4 ChIP-seq, Pol II ChIP-seq and ATAC-seq) are available at the NCBI GEO repository under accession number GSE162299, https://www.ncbi.nlm.nih.gov/geo/query/acc.cgi?acc=GSE162299 [44]. Publicly available RNA-seq data used in this study are deposited in the NCBI GEO repository with the accession number GSE88473 [45]. All scripts are available on GitHub, https://github.com/sblab-bioinformatics/G4_and_trancription [46] and Figshare [47].
