# Supplementary material for: Promoter G-quadruplex folding precedes transcription and is controlled by chromatin
Source: Genome Biol. 2021 May 7;22:143. doi: 10.1186/s13059-021-02346-7 (PMC8103603; doi:10.1186/s13059-021-02346-7)
Supplement: Supplementary file 1 — Additional file 1: Fig. S1. Endogenous G4 landscape in human K562 cells. Fig. S2. Induction of hypoxia decreases chromatin accessibility leading to G4 loss. Fig. S3. Hypoxia-induced chromatin compaction results in the reduction of promoter G4 folding in U2OS cells. Fig. S4. Response of endogenous G4s and Pol II following pre-treatment with G4-stabilising ligands before and after hypoxia induction. Fig. S5. BG4 immunofluorescence (IF) staining for G4s in DMSO- or pyPDS-treated cells upon hypoxia treatment. [file 13059_2021_2346_MOESM1_ESM.pdf]

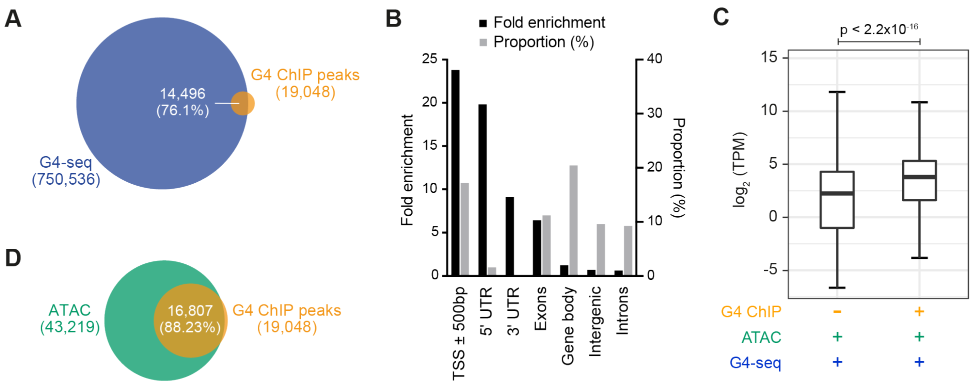

Fig. S1: Endogenous G4 landscape in human K562 cells. (A) Overlap of endogenous G4s (G4 ChIP peaks) found in K562 cells with sequence motifs that can fold into G4 structures *in vitro* (defined as G4-seq sites, see *ref 15*). **(B)** Enrichment and proportion of endogenous G4s by genomic feature. TSS, transcription start site; UTR, untranslated region. **(C)** Distribution of mRNA levels for genes with promoters in open chromatin (ATAC +) with an endogenous G4 (G4 ChIP peaks) or a G4 sequence motif that does not fold into a G4 (G4-seq sites) in K562 cells. $p < 2.2 \times 10^{-16}$, Wilcoxon test. TPM, transcripts per million. **(D)** Overlap of endogenous G4s (G4 ChIP-seq peaks) and open chromatin regions defined by ATAC-seq.

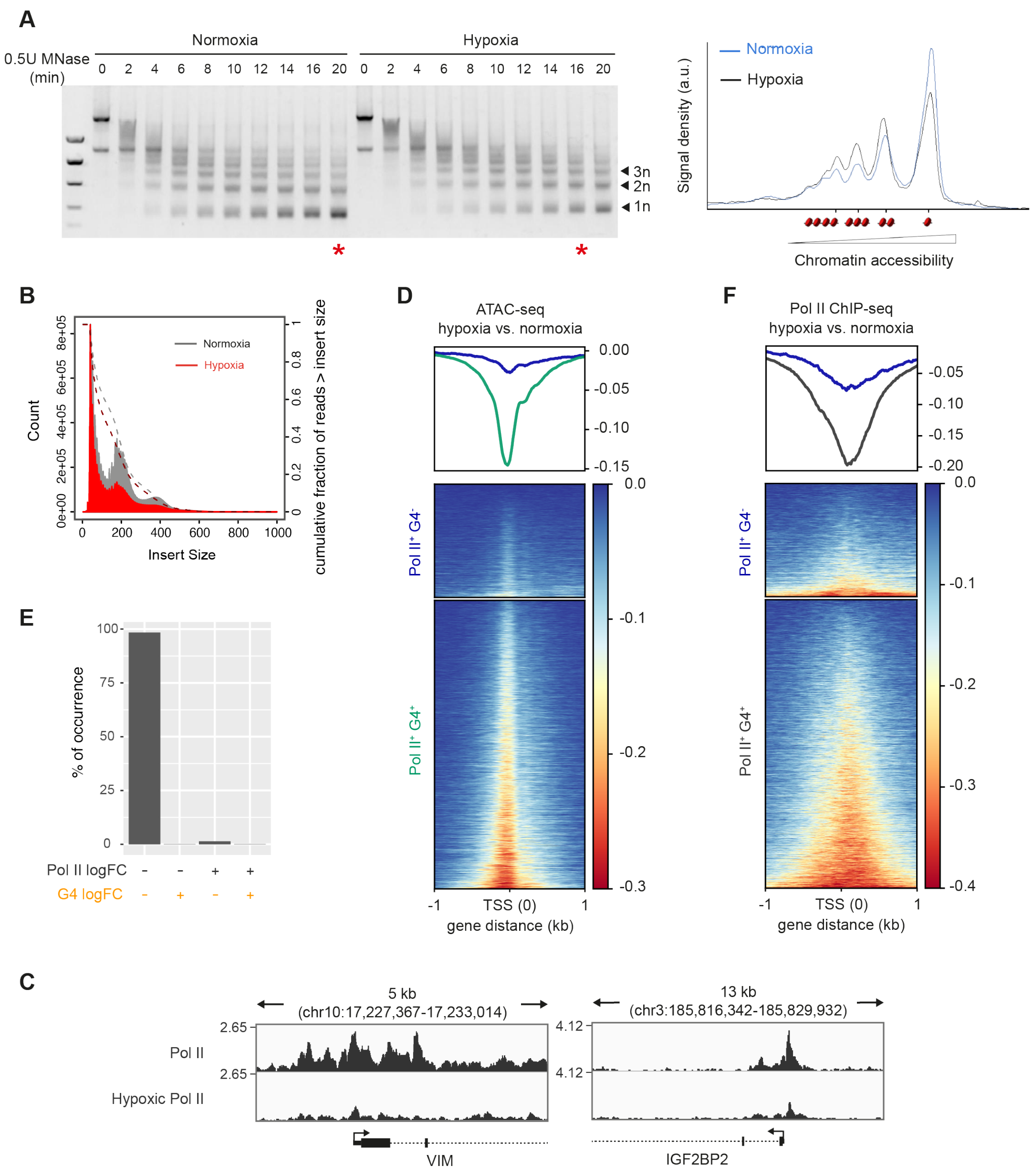

Fig. S2: Induction of hypoxia decreases chromatin accessibility leading to G4 loss. (A) Nucleosome distributions for cells cultured under normoxic (left) or hypoxic (right) conditions as assessed by micrococcal nuclease (MNase) digestion. 1n = mono-nucleosomes, 2n = di-nucleosomes, 3n = tri-nucleosomes. Asterisks indicate samples quantified by the graph on the right. Normoxia refers to cells cultured in 21% O_2 and hypoxia refers to cells exposed to 1% O_2 for 1 h. **(B)** Read counts and size distribution for different ATAC-seq fragment sizes for hypoxic (grey) and normoxic (red) chromatin. **(C)** Example genomic browser view showing

significantly reduced Pol II occupancy for cells cultured under hypoxic versus normoxic conditions. Genomic coordinates for *VIM* and *IGF2BP2* are indicated. **(D, F)** Signal differences in ATAC-seq signal or Pol II occupancy for promoters of active genes with or without a G4 (Pol II^+ $G4^-$ or Pol II^+ $G4^-$, respectively) under hypoxia or normoxia. Top panel, metagene plot of the normalised ATAC signal difference or Pol II ChIP-signal difference centered at the TSS in hypoxic versus normoxic condition. Bottom panel, data plotted for individual loci and represented by a heatmap plot. **(E)** Co-occurrence of loss of G4s and Pol II occupancy at promoters under hypoxic conditions. Genomic overlap of promoter G4s that have increases or reductions in G4 signal (G4 logFC + or – respectively), with increases or reductions in Pol II (Pol II logFC + or – respectively) at the same site graphed as percentage of co-occurrence.

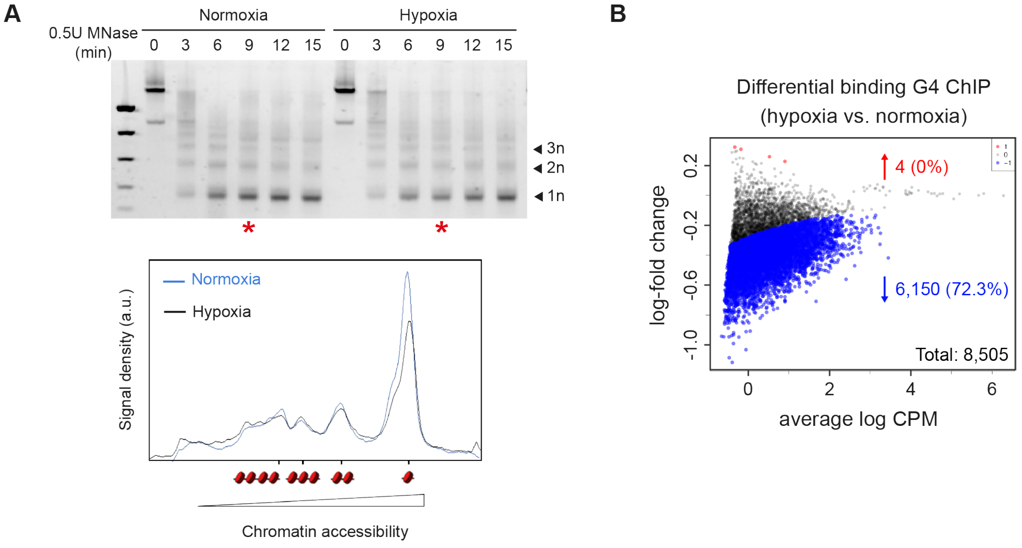

Fig. S3: Hypoxia-induced chromatin compaction results in reduction of promoter G4 folding in U2OS cells. (A) Nucleosome distributions for U2OS cells cultured under normoxic (left) or hypoxic (right) conditions as assessed by micrococcal nuclease (MNase) digestion. 1n = mono-nucleosomes, 2n = di-nucleosomes, 3n = tri-nucleosomes. Asterisks indicate samples quantified by the graph on the bottom. Normoxia refers to cells cultured in 21% O_2 and hypoxia refers to cells exposed to 1% O_2 for 1 h. **(B)** MA plot showing fold change in G4 ChIP-seq signal following hypoxia at promoters. Blue and red, sites with significantly reduced or increase signal respectively ($p < 0.05$). CPM, read count per million.

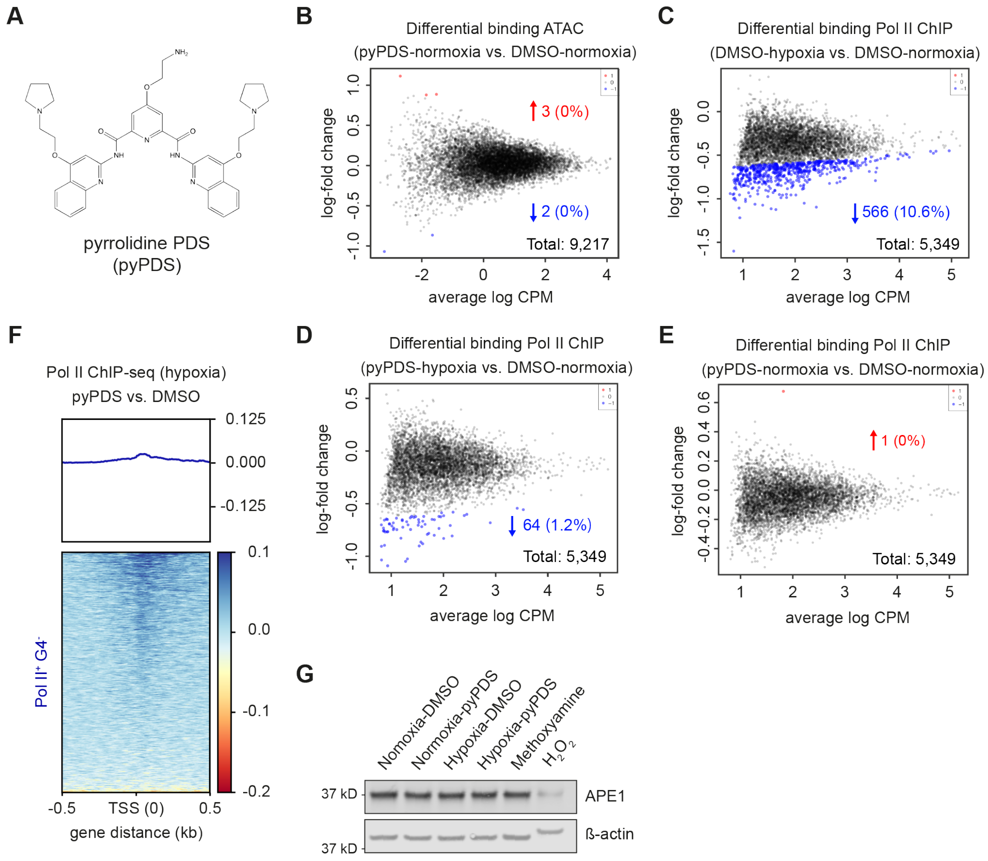

Fig. S4: Response of endogenous G4s and Pol II following pre-treatment with G4-stablising ligands before and after hypoxia induction. (A) Chemical structure of the G4-stabilising ligand, pyrrolidine PDS (pyPDS). **(B)** MA plot showing fold-change in ATAC-seq signal at promoters between pyPDS- and DMSO-treated cells under normoxic conditions. Significant increases or decreases ($p < 0.05$) in G4 ChIP-seq signal in pyPDS-treated cells relative to DMSO-treated cells are indicated in red and blue respectively. CPM, read count per million. **(C)** As in panel B but for changes in Pol II ChIP-seq signal ($p < 0.05$) between hypoxic and normoxic cells treated with control DMSO. **(D)** As in panel B but for changes in Pol II ChIP-seq signal for cells treated with pyPDS and subjected to hypoxia compared to DMSO-treated cells under normoxia. **(E)** As in panel B but for changes in Pol II ChIP-seq signal for cells treated with pyPDS or DMSO under normoxia. **(F)** Signal differences in Pol II occupancy for non-G4-marked promoters of active genes (Pol II$^+$ G4$^-$) under hypoxic condition treated

with DMSO or pyPDS. Top panel, metagene plot of Pol II signal centered at the TSS showing the signal difference between DMSO- and pyPDS-treated hypoxic cells. Bottom panel, data plotted for individual loci and represented by a heatmap plot. **(G)** Western blotting for APE1 under DMSO- or pyPDS-treated hypoxic and normoxic K562 cells. Methoxyamine and H_2O_2 treated cells represent negative and positive controls for APE1 respectively. Loading control, ß-actin.

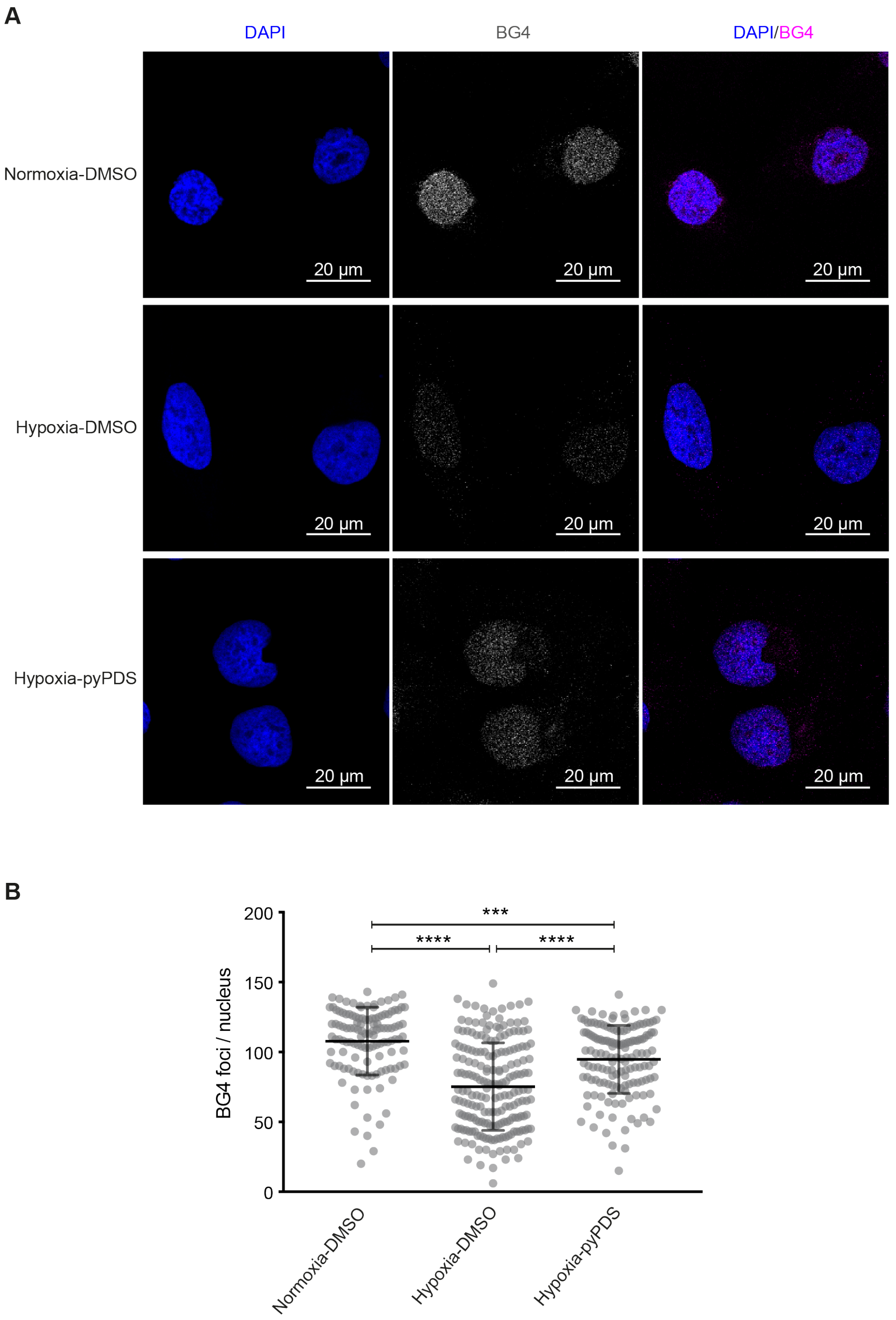

Fig. S5: BG4 immunofluorescence (IF) staining for G4s in DMSO- or pyPDS-treated cells upon hypoxia treatment. U2OS cells were fixed in paraformaldehyde, RNase A treated and stained with BG4 antibody. Confocal images were captured using a Leica SP5 microscope. **(A)** BG4 staining (grey/red) in the nuclei stained by DAPI (blue) of DMSO- or pyPDS-treated U2OS cells upon hypoxia treatment. DMSO-treated normoxic U2OS cells are controls. Scale bar = 20 µm. **(B)** Quantification of BG4 nuclear foci in individual cells. Each dot represents a

single nucleus. 150-200 nuclei were counted per condition and a one-way ANOVA test was performed for each condition across the mean values. Error bars represent the standard deviation (s.d.). ***: $p < 0.001$; ****: $p < 0.0001$.
