# Supplementary material for: Promoter G-quadruplex folding precedes transcription and is controlled by chromatin
Source: Genome Biol. 2021 May 7;22:143. doi: 10.1186/s13059-021-02346-7 (PMC8103603; doi:10.1186/s13059-021-02346-7)
Supplement: Supplementary file 2 — Additional file 2. Uncropped Western blots. [file 13059_2021_2346_MOESM2_ESM.pdf]

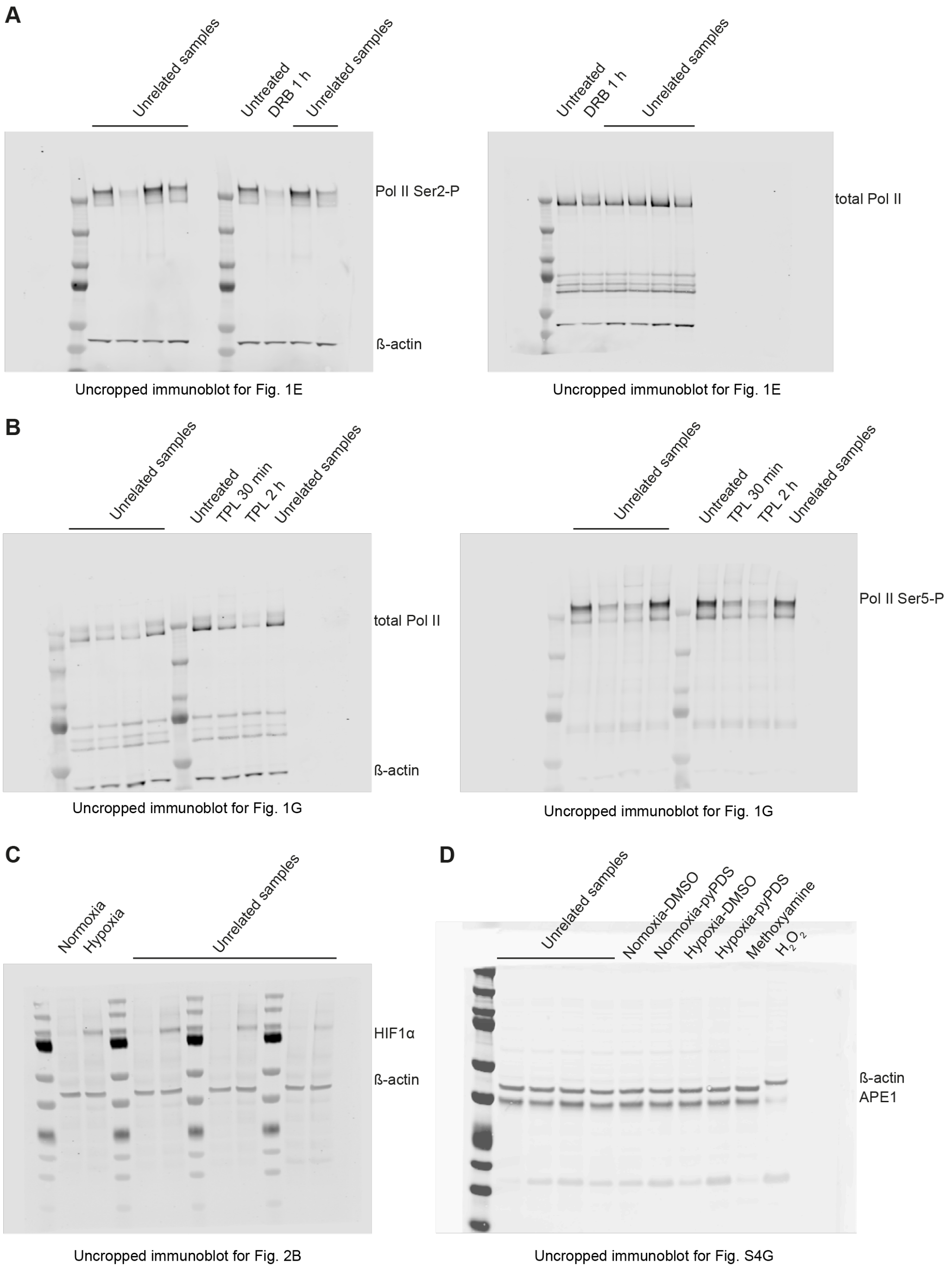

Fig. S6: Uncropped Western blots for **(A)** Fig. 1E, **(B)** Fig.1G, **(C)** Fig. 2B and **(D)** Fig. S4G.

Lanes labelled unrelated samples were not used as part of the main figures.
