# Supplementary material for: Promoter G-quadruplex folding precedes transcription and is controlled by chromatin
Source: Genome Biol. 2021 May 7;22:143. doi: 10.1186/s13059-021-02346-7 (PMC8103603; doi:10.1186/s13059-021-02346-7)
Supplement: Supplementary file 3 — Additional file 3. Review history. [file 13059_2021_2346_MOESM3_ESM.docx]

Review History

**First round of review**

**Reviewer 1**

**Are you able to assess all statistics in the manuscript, including the appropriateness of statistical tests used?** Yes, and I have assessed the statistics in my report.

**Comments to author:**

Shen et al have investigated the relationship between G-quadruplexes (G4s) and transcription by Pol II.

They have carried out genome-wide analysis of G4s and Pol II levels and ATAC-seq, and the results provide good evidence that these are positively correlated. Inhibiting pol II transcription by DRB or Triptolide has only a modest (and opposite!) effect on G4 levels, indicating that G4s are largely independent of Pol II initiation and occupancy. On the other hand, conditions that reduce G4s, like hypoxia, also cause a reduction of Pol II occupancy. As hypoxia causes some compaction of chromatin, the authors conclude that chromatin compaction is causing disruption of G4, which, in turn, leads to a reduction in Pol II levels. The authors have also investigated the effect of a G4-stabilising small molecule, pyPDS. pyPDS treatment causes an increase of both G4s and Pol II levels in hypoxia, further suggesting that G4 occupancy has a positive effect on pol II occupancy.

The data presented is convincing and the conclusions are generally supported by the data. In addition, the methods are appropriate, the paper is well-written and clear and the results will be of interest to others investigating the relationship between G4s and Pol II.

However, I have a couple of comments.

Using hypoxia to cause chromatin compaction leads to the problem that it could be something else that happens in hypoxia that is causing loss of G4s rather than compaction itself. I therefore think that the conclusion that only chromatin compaction is playing a role is overinterpretation of the data.

In addition, the molecular mechanisms that are operating are not at all clear to me-are the G4s recruiting proteins needed for transcription? If so, what does pyPDS do to binding of these proteins? If the authors think that Pol II subunits are binding to G4s, can they show this?

**Reviewer 2**

**Are you able to assess all statistics in the manuscript, including the appropriateness of statistical tests used? Yes, and I have assessed the statistics in my report.**

**Comments to author:**

The work entitled 'Promoter G-quadruplex folding precedes transcription and is controlled by chromatin' carried out in the Balasubramanian´s Lab deals with one of the major questions in the G4-research field. The current hypothesis states that G4 folding at promoters depends on active transcription, which leads to torsional stress and negative superhelicity. However, by means of well-done experimental strategies, the authors were able to collect data suggesting that G4 folding is coupled to the establishment of accessible chromatin and does not require active transcription. In this sense, the work represents a significant advance over previously published studies.

The design of study was appropriated, allowing answering the research question. The methods are appropriate to the aims of the study, they are well described, and proper controls are included. Results are clearly presented and have been statistically well-treated. In general, conclusions are supported by the experimental data shown.

Minor concerns:

The Background section is clear, accurately describing the current answers and hypothesis in the research field. The references have been properly selected; however, I recommend including a work on the role of CNBP in G4-folding recently appeared in Nucleic Acids Research (doi: 10.1093/nar/gkz527).

Regarding the results:

1- K562 human chronic myelogenous leukaemia cell line was used as a model system. Obtaining similar results in other cell lines may possibly strengthen the conclusions. Indeed, as authors mentioned, adenocarcinoma cells do not show G4 alterations following TPL treatment.

2- Genome browser views for the ATXN1L and FSCN1 promoter G4s are shown in Fig. 3E. Why the authors did select these genes among many others? A brief argumentation regarding this issue might improve the work.

**Authors Response**

**Point-by-point responses to the reviewers’ comments:**

1. Using hypoxia to cause chromatin compaction leads to the problem that it could be something else that happens in hypoxia that is causing loss of G4s rather than compaction itself. I therefore think that the conclusion that only chromatin compaction is playing a role is overinterpretation of the data.

We used acute hypoxia (1 hour) to achieve rapid chromatin compaction and assess the impact on promoter G4 formation. This short treatment time circumvents non-specific or downstream effects on G4s. We clearly demonstrate chromatin compaction at sites that show G4 loss and importantly have ruled out DNA damage as a possible cause of G4 loss in this system. G4s are only observed in accessible chromatin and earlier work has suggested that chromatin relaxation leads to increased G4 formation (doi: 10.1038/ng.3662). While we cannot rule out unknown factors that might influence G4 formation, the most parsimonious interpretation of the data is that chromatin compaction in acute hypoxia is a direct cause of G4 loss.

2. In addition, the molecular mechanisms that are operating are not at all clear to me - are the G4s recruiting proteins needed for transcription? If so, what does pyPDS do to binding of these proteins? If the authors think that Pol II subunits are binding to G4s, can they show this?

G4s have been shown to interact with multiple proteins involved in the transcriptional process, which includes transcription factors and RNA polymerase subunits (doi: 10.1038/s41467-018-04084-0). To elaborate this further, we have added the following text to the discussion on page 9, line 189-190: “Pol II recruitment at promoter G4s might be mediated by TF binding [8–10], though it is also possible that G4s interact directly with subunits of RNA Pol II [34].”

In our experiments, the presence of pyPDS has allowed retention of Pol II at promoter G4s, which suggests that ligand binding does not perturb Pol II occupancy. The general impact of ligand binding on G4-protein interactions has not been systematically explored, but several reports provide evidence that small molecules can co-bind to G4 structures without perturbing protein interactions (doi: 10.1021/jacs.0c10792, doi.org/10.1073/pnas.1603724113, doi: 10.1093/nar/gkv051 and doi: 10.1002/anie.201408113).

Reviewer #2:

3. Minor concerns: The Background section is clear, accurately describing the current answers and hypothesis in the research field. The references have been properly selected; however, I recommend including a work on the role of CNBP in G4-folding recently appeared in Nucleic Acids Research (doi: 10.1093/nar/gkz527).

We now include the mentioned work in the Background section on page 3, line 45-47 to state: “Alternatively, TF recruitment to G4s could enhance transcription by unfolding the G4 structure, as has been suggested for CNBP [11].”

4. Regarding the results: K562 human chronic myelogenous leukaemia cell line was used as a model system. Obtaining similar results in other cell lines may possibly strengthen the conclusions. Indeed, as authors mentioned, adenocarcinoma cells do not show G4 alterations following TPL treatment.

We have carried out additional experiments and now provide additional experimental data confirming the loss of endogenous G4s following hypoxia-driven chromatin compaction in the human osteosarcoma cell line, U2OS. This strengthens our key conclusion that formation of promoter G4s is controlled by chromatin accessibility. We have appended these data as a new supplementary figure and added the following text on page 7, line 135-138: “We next validated the general principle of G4 loss upon hypoxia-associated chromatin compaction by using an additional unrelated cell line. Acute hypoxia in human osteosarcoma U2OS cells also induced chromatin compaction and led to a reduction of G4 signal intensity at promoters (p < 0.05; 6,150/8,505 down, 72.3%; Supplementary Fig. 3).”

5. Genome browser views for the ATXN1L and FSCN1 promoter G4s are shown in Fig. 3E. Why the authors did select these genes among many others? A brief argumentation regarding this issue might improve the work.

There was no particular reason for choosing *ATXN1L* and *FSCN1*, which were simply representative examples. We have now replaced the *ATXNL1* and *FSCN1* with *BRD4* and *CBX1* in Fig. 3E as genes of particular interest that are involved in the regulation of chromatin. Bromodomain protein BRD4 recognises acetyl-lysine of histones and plays an important role in regulating transcription and chromatin (doi: 10.1016/S0014-5793(01)03309-9; doi: 10.1016/j.celrep.2018.10.003). CBX1 encodes non-histone protein Chromobox protein homolog 1, which is a component of heterochromatin proteins HP1 (doi: 10.1186/gb-2006-7-7-228). We have altered the results section to reflect this change on page 8, line 161-163 to state: “These findings are exemplified by genome browser views for the promoters of the chromatin regulators *BRD4* and *CBX1* (Fig. 3E).”

**Second round of review**

**Reviewer 1**

The authors have not fully addressed my concerns. At the very least they should include the discussion of why they consider that chromatin compaction is the only thing affecting G4s when cells are hypoxic in the paper itself. In addition, the authors should make more comprehensive reference to other papers on the relationship between transcription and G4s.

**Reviewer 2**

Most the concerns have been satisfactorily resolved.

**Authors Response**

**Point-by-point responses to the reviewers’ comments:**

Reviewer 1:

The authors have not fully addressed my concerns. At the very least they should include the discussion of why they consider that chromatin compaction is the only thing affecting G4s when cells are hypoxic in the paper itself. In addition, the authors should make more comprehensive reference to other papers on the relationship between transcription and G4s.

To address the referee’s concern, we now provide a more detailed discussion on page 9: “We use acute hypoxia to achieve rapid chromatin compaction with a short treatment time to circumvent non-specific or downstream effects on G4 formation. Endogenous G4 structures are only observed in accessible chromatin and chromatin relaxation by histone deacetylation leads to increased G4 folding [5]. Recent imaging experiments in neuronal cells have also demonstrated that G4 folding requires chromatin accessibility [34]. While we cannot rule out unknown factors that might further influence G4 formation in hypoxia, the most straightforward interpretation of the data is that chromatin compaction in acute hypoxia leads to G4 loss.”
